# NK cell activity in the tumor microenvironment

**DOI:** 10.3389/fcell.2025.1609479

**Published:** 2025-05-30

**Authors:** A. V. Kuznetsova, X. A. Glukhova, I. P. Beletsky, A. A. Ivanov

**Affiliations:** ^1^ Laboratory of Molecular and Cellular Pathology, Russian University of Medicine, Ministry of Health of the Russian Federation, Moscow, Russia; ^2^ Laboratory of Regenerative Biology, Koltzov Institute of Developmental Biology, Russian Academy of Sciences, Moscow, Russia; ^3^ Onni Biotechnologies Ltd., Helsinki, Finland

**Keywords:** human natural killer cells, tumor microenvironment, extracellular matrix, new targets, anti-NK cell signaling

## Abstract

The formation of an immunosuppressive tumor microenvironment (TME) impairs natural killer (NK) cell infiltration and persistence within tumor tissue and significantly diminishes NK-mediated cytotoxicity. This presents a substantial barrier to the efficacy of NK cell therapy in solid tumors. Current strategies aim to overcome immune evasion by enhancing NK cell recognition and cytotoxicity, while promoting their persistence, infiltration, and resistance to the TME. This review focusses on the biophysical characteristics of TME and specific components of the extracellular matrix (ECM) that affect NK cell activity, with the goal of identifying therapeutic approaches to modulate the TME and create a supportive niche for adaptive immune cell function. Advancements in interdisciplinary collaborations integrating oncology, cell biology, physics, engineering, materials science, and nanotechnology are crucial in advancing therapeutic strategies targeting ECM rigidity and mechanotransduction signaling pathways.

## 1 Introduction

Natural killer (NK) cells are a tempting alternative and/or perhaps even a necessary complement to T-cell anti-cancer therapy. Unlike T- and B-cells, they rapidly attack target cells without prior sensitisation or antigen recognition. These cells exert immune defence against infected or transformed cells through their direct effector functions (cytotoxicity and production of cytokines including interferon-γ (IFN-γ), tumor necrosis factor-α (TNF-α), CCL5 etc.) and immunoregulatory functions (e.g., interaction with dendritic cells (DCs)) (Bi and Wang, 2020). Their functions are regulated by numerous activating and inhibitory cell membrane receptors (Sivori et al., 2019; Liu and Sun, 2021; Cózar et al., 2021).

Advances in the study of NK cells have moved research, particularly those developing appropriate CAR or TCR constructs, into early phase clinical trials that have shown a favorable safety profile and promising responses (Page et al., 2024). However, these studies also demonstrated disease recurrence in many patients after an initial reduction in tumor burden, which may be partly explained by tumor escape and depletion of target NK cells.

One of the barriers to the use of NK-cell therapy in solid tumors is the immunosuppressive effect of the tumor microenvironment (TME), which reduces NK cell infiltration and persistence in tumor tissue and strongly attenuates NK-mediated cytotoxicity (Vyas et al., 2022). Furthermore, achieving successful outcomes with NK-cell therapy depends not only on the ability of immune cells to migrate and persist in TME, but also on their ability to remain functional despite an immunosuppressive environment. Consequently, various strategies have been explored to potentially overcome this barrier, including the use of chemokine networks to effectively recruit NK cells into the TME and the enhancement of NK cell properties to resist immunosuppression (Ben-Shmuel et al., 2020). These strategies are directed at the NK cells themselves. However, growing evidence of the essential role of the extracellular matrix (ECM) in tumor-immune interactions, in addition to its well-established role in the tumor regulation of the tumor itself, suggests that the ECM is an equally relevant target for anti-cancer immunotherapy (Du et al., 2024; Miao et al., 2024; Lightsey and Sharma, 2024). It has been shown that tumor cell-induced changes in the ECM can affect both the ability of immune cells to migrate and infiltrate tumor tissue, as well as their phenotype and metabolism. In particular, NK cells infiltrating the TME have been shown to exhibit reduced expression of activating receptors (including DNAM1, NKp80, NKp30 and CD16), which impairs their cytotoxicity, and increased expression of inhibitory molecules known as immune checkpoints, including programmed cell death protein 1 (PD-1), T-cell activation, increased late expression (TACTILE/CD96), T-cell immunoglobulin and mucin-domain-containing protein 3 (TIM3), and T-cell immunoglobulin and ITIM domain (TIGIT), among others, which cause NK cell exhaustion upon binding to appropriate ligands on tumor cells (Sivori et al., 2019; Sanchez-Correa et al., 2019; Cózar et al., 2021; Lian et al., 2021; Ran et al., 2022; Berrien-Elliott et al., 2023; Closset et al., 2023). Thus, identifying and overcoming barriers in the TME of solid tumors is crucial to improving NK cell delivery and optimising their anti-tumor functions, which is essential for enhancing the efficacy of NK cell therapy (Lightsey and Sharma, 2024).

The formation of an immunosuppressive TME is a complex and multifactorial process. In general, NK cell dysfunction, which facilitates tumor escape from immune surveillance, is related to TME properties such as: (1) physical characteristics of the TME, pertained including changes in macromolecule components and degradation enzymes, increased stiffness and decreased porosity of the ECM, and decreased mechanical stiffness and the acquisition of softness by tumor cells; (2) metabolic characteristics of the TME, such as hypoxia and acidity, nutrient deficiencies (e.g., glucose, amino acids), accumulation of nitric oxide, hydrogen peroxide, and specific metabolites (e.g., lactate, adenosine); and (3) aberrant activation of signaling pathways (e.g., transforming growth factor-beta (TGF-β), STAT3) (Zhang et al., 2024; Du et al., 2024; Lightsey and Sharma, 2024).

While the impact of the metabolic features of TME on NK cell effector functions has been discussed in more detail elsewhere (Noman et al., 2015; Zhou et al., 2023; Portale and Di Mitri, 2023; Miao et al., 2024), this mini-review focuses on the biophysical features of the TME and specific ECM proteins that affect NK cell activity, with the aim of identifying therapeutic strategies to alter the TME and create a favorable niche for adaptive immune cell functioning.

## 2 NK cells. Basic instinct

NK cells are formed from hematopoietic stem cells (HSCs) primarily in the bone marrow and then begin the maturation process before heading to the periphery for immune control. NK cell maturation progresses through several stages in the bone marrow and other sites (e.g., fetal liver, thymus, and tonsils), where HSCs differentiate first into common lymphoid precursors (CLPs) and then into NK cell precursors (NKPs). The development of NK cells from HSCs is regulated by a set of cytokines. Among the many receptors, the sequential expression of which determines the functional maturation of NK cells, two reference molecules are notable: CD122 and CD56 (Nitta et al., 1989; Huntington et al., 2009). Expression of CD122, the common β-chain for IL-2R and IL-15R, on NKPs is critical for NK cell commitment. IL-15 is required for the differentiation of NKPs into mature NK cells. IL-15 induces activation of the JAK-STAT5 pathway, which triggers the expression of STAT-targeting sequences involved in NK cell development, survival, proliferation, and function (Lin et al., 2017; Witalisz-Siepracka et al., 2019). It has been shown in mice that the growth of breast cancer, colorectal cancer, and melanoma cells can suppress functional maturation of NK cells in the bone marrow by interrupting the IL-15 signaling pathway (Richards et al., 2006). CD56, a member of the immunoglobulin superfamily involved in both homophilic and heterophilic interactions, appears at the final stages of NKP differentiation into NK cells. Depending on the level of CD56 expression, two stages of NK cell maturation are distinguished: the CD56bright stage and the CD56dim stage. CD56bright NK cells are considered immature and can differentiate into CD56dim NK cells with acquisition of CD16.

Conversely, CD56dimCD16+ NK cells can switch to a CD56brightCD16− phenotype and proliferate in response to 4-1BBL + IL-12, as has been shown in NK cells from patients with renal or ovarian cancer (Dowell et al., 2012; Bi and Wang, 2020; Ran et al., 2022). It is generally accepted that CD56bright NK cells specialise in producing pro-inflammatory cytokines, and whereas CD56dim NK cells are characterised as more cytotoxic (Ran et al., 2022; Miao et al., 2024). Specifically, IL-2 production has been shown to be stimulated by the interaction of CD56 on NK cells with FGFR1 on T cells, and CD56-dependent cytotoxicity has been shown to be mediated by phosphorylation of proline-rich tyrosine kinase 2 (Pyk2) (Kos and Chin, 2002; Gunesch et al., 2020).

Under normal physiological conditions, the effector functions of NK cells are inhibited by major histocompatibility complex class I molecules (MHC-I)/human leukocyte antigen class I (HLA-I) molecules, which are expressed on the surface of healthy cells. HLA class I molecules act as ligands for key inhibitory NK cell receptors, such as members of the killer cell immunoglobulin-like receptor (KIR) family and the CD94/natural killer group (NKG) receptor 2 A (NKG2A) heterodimer, promoting NK cell immune tolerance (Garrido and Aptsiauri, 2019; Zhang et al., 2024).

NK cells can control tumor growth by recognising and destroying abnormal cells that lack HLA class I expression. They are particularly effective in controlling circulating tumor cells in hematological malignancies and in restricting tumor metastasis. However, only a small fraction of NK cells typically infiltrate solid tumors and are mostly confined to the stroma at the tumor’s invasive margin (Garrido and Aptsiauri, 2019; Bunting et al., 2022; Lightsey and Sharma, 2024). The efficacy of NK cell-mediated tumor cell killing is thought to correlate with the pathways of metastatic dissemination. Notably, the destruction of HLA class I-negative metastatic cells by NK cells is more intense when the metastatic cells spread via the bloodstream (Garrido and Aptsiauri, 2019).

Loss of HLA-I expression in certain malignancies is accompanied by overexpression of HLA-E, which is associated with a poor outcome (Zhang et al., 2024). HLA-E is a ligand for both an inhibitory receptor (CD94/NKG2A) and an activating receptor (CD94/NKG2C) on NK cells, which recognise partially overlapping but distinct epitopes of HLA-E. Although both NKG2A and NKG2C bind to HLA-E, the activating receptor exhibits significantly lower affinity for its ligand (Braud et al., 1998; Wada et al., 2004; Kaiser et al., 2005). Binding of CD94/NKG2A to HLA-E molecules expressed by solid tumors results in inhibition of NK cell cytotoxicity. This has been demonstrated in various tumor cell lines derived from melanoma, acute myeloid leukemia (AML), osteosarcoma and Ewing’s sarcoma (Berezhnoĭ et al., 2009; Kamiya et al., 2019; Zhang et al., 2024).

**FIGURE 1:**
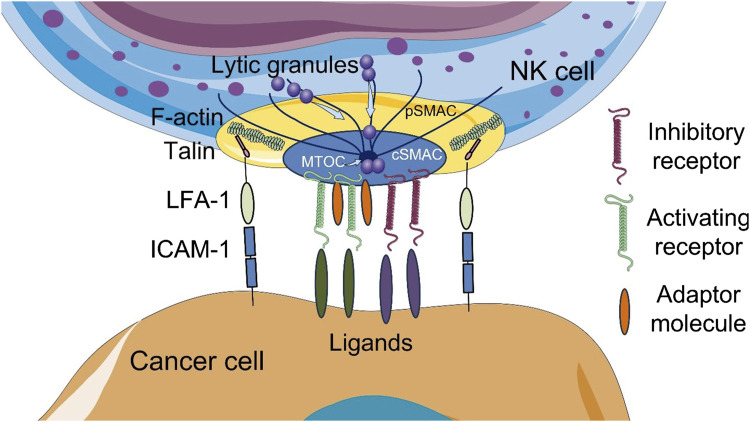
Schematic illustration of the cytotoxic NK cell immune synapse (NKIS). The NKIS contains two supramolecular activation clusters (SMACs): central SMAC (cSMAC) and peripheral SMAC (pSMAC). Lytic granules are transported to the synapse along the microtubules. Talin, a mechanosensitive adhesion protein, directly links integrins to filamentous actin (F-actin). ICAM-1, intercellular adhesion molecule 1; LFA-1, lymphocyte function-associated antigen 1; MTOC, microtubule-organizing center polarity. The figure contains modified images from Servier Medical Art (https://smart.servier.com) licensed by the Creative Commons Attribution CC BY 4.0 International License (https://creativecommons.org/licenses/by/4.0/).

To initiate contact between an NK cell and a susceptible target cell, surface receptors on NK cells interact with appropriate ligands, triggering the formation of a specific structure known as the NK cell immunological synapse (NKIS) (Figure 1). The NKIS contains regions with different protein composition and actin dynamics known as supramolecular activation clusters (SMACs). The central SMAC (cSMAC) contains inhibitory and activating receptors, lytic granules and signaling molecules, while the peripheral SMAC (pSMAC) contains integrins (lymphocyte function-associated antigen (LFA)-1 (CD11a/CD18), LFA-2/CD2, Mac-1 (CD11b/CD18)) and cytoskeletal components, such as filamentous actin (F-actin), which mediate adhesion (Orange et al., 2003; Roda-Navarro and Reyburn, 2007; Topham and Hewitt, 2009; Prager and Watzl, 2019; Ben-Shmuel et al., 2021).

The main activating receptors for NK cells are the natural cytotoxicity receptors (NCRs): NKp30 (NCR3), NKp44 (NCR2) and NKp46 (NCR1), as well as CD16a and NKG2D. Other molecules such as DNAM1, 2B4, and NKp80 enhance NK cell activity, acting primarily as coreceptors (Li and Sun, 2018; Cózar et al., 2021). Activated NK cells can directly destroy target tumor cells by releasing cytoplasmic lytic granules containing perforin and granzyme. NK cells also express members of the TNF family proteins such as Fas ligand (FasL) or TNF-related apoptosis-inducing ligand (TRAIL), which induce tumor cell death by interacting with their respective receptors. In addition, antibody-dependent cellular cytotoxicity (ADCC), mediated by the CD16a receptor (FcγRIIIa), can induce NK-mediated death of tumor target cells coated with antibodies bound to antigens on their surface (Liu and Sun, 2021; Ran et al., 2022).

## 3 Lost in penetration

**FIGURE 2:**
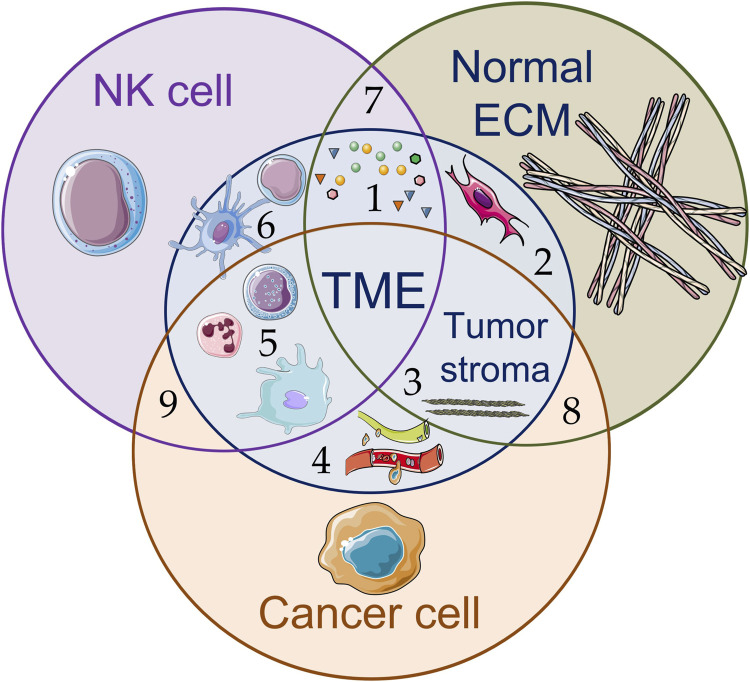
Schematic illustration of various components of the tumor microenvironment (TME) (1–6) and interactions between NK cells, cancer cells, and components of the extracellular matrix (ECM) (7–9): (1) signaling molecules, including soluble tumor-derived factors and ligands (growth factors, chemokines, proangiogenic and anti-inflammatory cytokines, metabolites, etc.), inhibit NK cell effector function, support tumor development (tumor growth, metastasis) and stimulate (2) resident fibroblasts and cancer-associated fibroblasts to accumulate rigid and disordered ECM, leading to (3) increased stiffness of fibrillar structures and decreased porosity of the tumor stroma; (4) blood and lymphatic vessels; (5) myeloid cell infiltration promote functional suppression of effector cells (such as NK cells and CD8^+^ cytotoxic T-lymphocytes (CTLs)); (6) dendritic cells induce anticancer responses of CTLs and stimulate CD4^+^ T-cells to produce signaling molecules. The figure contains modified images from Servier Medical Art (https://smart.servier.com) licensed by the Creative Commons Attribution CC BY 4.0 International License (https://creativecommons.org/licenses/by/4.0/).

NK cells require close proximity or direct contact with target cells to carry out their effector functions. Accordingly, in solid tumors, NK cells must overcome the physical and biochemical barriers created by ECM and TME cells to infiltrate the tumor, reach cancer cells and induce their destruction (Closset et al., 2023) (Figure 2). Poor tumor infiltration by NK cells is a well-known phenomenon and represents a significant obstacle and challenge of contemporary adaptive cell therapies, ultimately limiting their efficacy.

NK cells need to perceive and respond to their physical environment in order to reach and destroy cancer cells. Tissue stiffness score is one of the most important biomechanical characteristics of solid tumors. The stiffness of ECM and cancer cells is controlled and recognised in part by cellular mechanosensitive ion channels such as the PIEZO (mechanoactivated cation channels) family, the TREK/TRAAK two-pore potassium channels, the hyperosmolar calcium-permeable OSCA/TMEM63 channels and the transmembrane channel-like (TMC-like) 1/2 channels. The role of mechanosensing in NK-cell killing efficiency have been demonstrated in the studies describing mechanosensitive ion channels in NK cells (Schulte-Mecklenbeck et al., 2015; Zöphel et al., 2023; Yanamandra et al., 2024). As shown in Yanamandra et al., the PIEZO1 ion channel is the predominant mechanosensitive channel expressed in human NK cells; it is present at the plasma membrane and regulates the responsiveness of NK cells to target cell stiffness. Activation of PIEZO1 significantly enhances cancer cell elimination, increases NK cell degranulation, and improves NK cell infiltration in three-dimensional collagen matrices.

Remarkably, the durations required for NK-cell killing and detachment are significantly shortened for stiffened tumor cells than for their softer counterparts (Yanamandra et al., 2024). A number of studies using atomic force microscopy have shown that cancer cells are softer than their normal non-malignant counterparts. For example, cervical cancer cells have an elastic modulus of ∼2 kPa, which is lower than that of normal human cervical epithelial cells (elastic modulus E ∼4–5 kPa) (Hayashi and Iwata, 2015). As another example, ovarian cancer cells have Young’s modulus in the range of 0.5–1 kPa, while their non-malignant counterparts have a stiffness of about 2 kPa (Xu et al., 2012). Notably, even among malignant cells stiffness can vary, with softer cancer cells displaying increased tumorigenicity, metastatic potential, and stemness. Cell stiffness affects the efficiency of cell destruction by immune cells. Specifically, NK cell cytotoxicity is reduced against softer tumor cells and enhanced against stiffer ones. Interestingly, the relationship between NK cell degranulation activity and target cell stiffness follows a bell-shaped curve, peaking at approximately 200 kPa, and is regulated by the interaction between activating NKG2D receptors and their MICA ligands (Rianna et al., 2020; Mordechay et al., 2021; Lv et al., 2021; Yanamandra et al., 2024). Target cell stiffness plays a crucial role in NKIS formation: polarisation of the microtubule organisation centre and lytic granules in NK cells is impaired when engaging with soft targets, resulting in unstable immune synapses (Friedman et al., 2021). Thus, cell softening may be regarded as a way by which malignant cells evade immune surveillance.

In addition, biophysical characteristics of the tumor ECM may influence the cytotoxic synapse. Cancer cells attached to rigid substrates have been shown to exhibit increased cellular tension, which facilitates perforin-mediated pore formation by cytotoxic immune cells (Cho and Doh, 2024). NK-92 cells demonstrate significantly higher cytotoxicity against various human cancer cells cultured on nanoribbon surfaces compared to those on flat surfaces. In cancer cells siRNA-mediated knockdown of Rho-associated coiled-coil forming kinase (ROCK), which regulates cytoskeletal reorganization under cellular stress results in reduced NK cell cytotoxicity against cancer cells on nanoribbon surfaces (Cho et al., 2023). In line with this, NK cells show greater degranulation against hard hydrogel beads coated with anti-NKp30 and anti-LFA-1 antibodies than against soft hydrogel beads with the same coating, further underscoring the role of cancer cell stress in NK-mediated cytotoxicity (Friedman et al., 2021).

Physical barriers to cytotoxic immune cell infiltration, summarised as “lost in penetration”, include the increased density, rigidity, cross-linking, and alignment of the fibrillar ECM structures. Cell migration along or through the three-dimensional ECM depends on the physicochemical balance between ECM density, matrix pore size, and cell deformability (size and mechanical properties of their nucleus). The rate and limits of cell migration in the interstitium are further modulated by the ability to degrade ECM using proteolytic enzymes, mainly matrix metalloproteinases (MMPs). However, because the nucleus is the main migration-limiting cellular compartment, successful passage through the dense ECM requires nuclear deformation via both integrin-mediated traction force and actomyosin-driven contractility (Wolf et al., 2013; Kalukula et al., 2022; Wong and Ding, 2023; Cho and Doh, 2024). In contrast to migration of solid tumor cells, which form prominent protrusions and spindle-shaped morphology and display strong adhesion to ECM and proteolytic tissue remodeling (pericellular collagenolysis), the interstitial movement of leukocytes is marked by an ellipsoidal, rapidly deforming morphologies with small protrusions, weak adhesion and no proteolysis (Wolf et al., 2013).

The increased density of fibrillar structures of the tumor ECM is determined primarily by the orientation and density of collagen fibres and the spacing between them, all of which influence the distribution and migration of cytotoxic lymphocytes, CD8^+^ cytotoxic T lymphocytes (CTLs) and NK cells within the tumor stroma (Du et al., 2024; Cho and Doh, 2024). Cytotoxicity analysis conducted using 3D microfluidic chips filled with different concentrations of collagen and encapsulated tumor cells, mimicking the complex TME, has shown that NK-92 cell infiltration into collagen gels decreases significantly as collagen density increases (Park et al., 2019). The increased density and cross-linking of ECM reduce its porosity, which is critical in limiting cytotoxic lymphocyte infiltration into the tumor stroma. As demonstrated by Wolf et al., MMP-independent cell migration declined as linear function of pore size and deformation of the nucleus, with tumor cell motility ceasing at a collagen pore size of 7 μm^2^, T cells at 4 μm^2^, and neutrophils at 2 μm^2^ (Wolf et al., 2013).

The orientation and density of collagen fibres is dictated among others by their alignment and straightness, with bundles of straightened and aligned collagen fibres orientated perpendicular to the tumor border. This is achieved, in particular, by binding of the discoidin domain receptor 1 (DDR1), a collagen receptor with tyrosine kinase activity, of tumor cells to the fibrillar collagen of the TME. Mechanistically, binding of the extracellular domain of DDR1 (DDR1-ECD) to collagen enforces alignment of collagen fibres and restricts infiltration of immune cells (T cells, NK cells and DCs). DDR1-ECD-neutralising antibodies disrupt collagen fibre alignment, enhance immune cell infiltration and suppress tumor growth (Conklin et al., 2011; Sun et al., 2021; Li T. et al., 2024). At the same time, reducing of type I collagen in pancreatic cancer accelerates tumor progression by activating CXCL5 in cancer cells, thereby recruiting myeloid-derived suppressor cells (MDSCs) that suppress cytotoxic CD8^+^ T cells in both murine and human models (Chen et al., 2021). MDSCs are also known inhibit NK cell antibody-dependent cellular cytotoxicity (ADCC) via nitric oxide secretion, and T regulatory (Treg) cells can directly or indirectly suppress NK cell function through IL-10 secretion (Cózar et al., 2021).

## 4 TME-mediated anti-NK cell signaling

**FIGURE 3:**
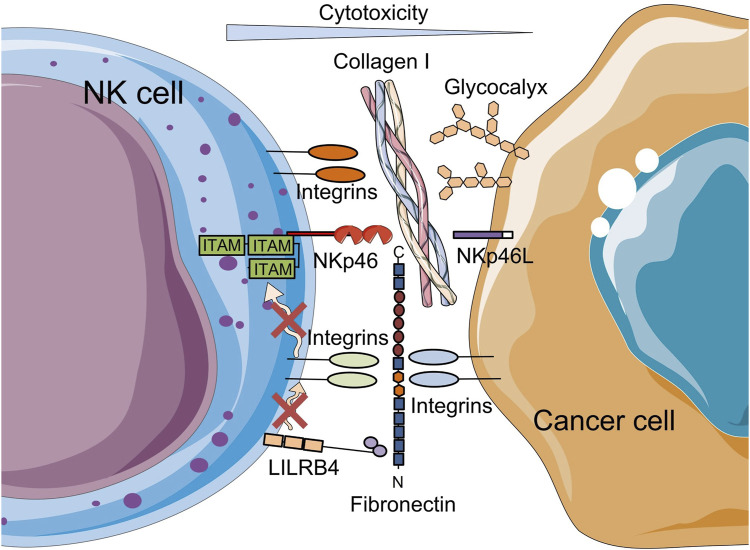
Schematic representation of the reduction of NK cell-mediated cytotoxicity by ECM components. On the one hand, the binding of type I collagen to NK cell receptors, such as integrins; on the other hand, increased glycocalyx thickness, a dense layer of glycosylated proteins and lipids, on tumor cells that protect tumor cells from interaction with immune cell receptors leads to a decrease in NK cell cytotoxicity. Fibronectin on cancer cells attenuates NK cytotoxicity through LILRB4. The figure contains modified images from Servier Medical Art (https://smart.servier.com) licensed by the Creative Commons Attribution CC BY 4.0 International License (https://creativecommons.org/licenses/by/4.0/).

In addition to collagen, other ECM components, such as fibronectin, laminin, elastin, and glycosaminoglycans (GAGs), are involved in the modulation of TME and cellular stiffness (Vogel, 2018). At the same time, these ECM components influence NK cell function through direct receptor stimulation (Bunting et al., 2022) (Figure 3). Collagen, like other ECM proteins, can mediate specific signaling pathways by binding to NK cell receptors such as integrins, DDRs, leukocyte-associated immunoglobulin-like receptor-1 (LAIR-1) and others. LAIR-1 and integrin expression (primarily integrin alpha M (ITGAM/CD11b), integrin alpha 2 (ITGA2/CD49b) and integrin beta-2 (ITGB2/CD18)), which interact with collagen I, collagen III and elastin has been shown to reduce NK cell cytotoxicity, while increasing their production of chemokines and cytokines when infiltrating peripheral tissues (Bunting et al., 2022; Vyas et al., 2022). Similarly, a study investigating interactions between tumor cells and the bone matrix in the context of bone metastatic breast cancer showed that collagen mineralization alters sialoglycan and mucin O-glycan expression, increasing the glycocalyx thickness of cancer cells. These changes, in turn, support evasion of NK cell-mediated cytotoxicity (Park et al., 2024). Since typical immune receptors extend only 10–20 nm from the effector cell surface, the thickness of the cancer cell glycocalyx, which can exceed 100 nm, becomes a key physical barrier to receptor-mediated interactions that are obligatory for cell-mediated cytotoxicity (Park et al., 2024).

Another important ECM component is laminin. To date, 16 isoforms of laminin have been identified. Each isoform consists of disulfide-linked heterotrimers formed from different combinations of α (1–5), β (1–3) and γ (1–3) chains (Nonnast et al., 2024). Analysis of The Cancer Genome Atlas (TCGA) database revealed a positive correlation between mRNA levels of laminin γ2 (monomeric laminin-332) and TGF-β1 in tumor cells from various types of human carcinomas, including non-small cell lung cancer and esophageal carcinoma (Li et al., 2021). Nearly all leukocyte subpopulations express specific laminin receptors. Laminins function as ligands that bind these receptors at the cell membrane, initiating signal transduction that modulates leukocyte function and migration (Simon and Bromberg, 2017). Laminins can bind NK cell receptors such as integrins and non-integrin receptors including proteoglycans and the high-affinity laminin receptor 1 (LAMR1) (Lesot et al., 1983). Although inhibition of NK cell cytolytic activity by laminin was demonstrated more than 40 years ago, more recent evidence suggests that laminin exerts only a modest influence on NK cell function, particularly degranulation and IFN-γ expression (Hiserodt et al., 1985; Bunting et al., 2022).

Fibronectin, another prominent ECM component, is abundant in the TME, and promotes tumor metastasis and immune evasion (Lin et al., 2019). Through canonical receptors such as α5β1 and αvβ3 integrins expressed on various cell types, including leukocytes, fibronectin enhances cell adhesion and regulates cellular function. Cross-linking of fibronectin to α4β1 and α5β1 integrins has previously been shown to enhance NK cell-mediated ADCC, suggesting a co-activating role for these integrins (Palmieri et al., 1995). However, these integrins may also serve as co-inhibitory receptors. Notably, the 30 kDa N-terminal domain of fibronectin has been identified as a ligand for the inhibitory NK cell receptor, immunoglobulin-like transcript 3/leukocyte immunoglobulin-like receptor B4 (ILT3/LILRB4) (Itagaki et al., 2023) (Figure 3). As a myeloid immune checkpoint, LILRB4 is expressed across a wide range of immune cell types including DCs, monocytes, macrophages, mast cells, B cells, NK cells and T cells (Sharma et al., 2021). Itagaki et al. showed that LILRB4 can bind fibronectin on target cells in *trans*-mode together with integrins in *cis*-mode and provide an inhibitory signal to NK cells, and thereby attenuating their cytotoxicity within the TME. Thus, LILRB4 and integrins may function as co-inhibitory receptors (Itagaki et al., 2023).

Glycosaminoglycans (GAGs) present in nearly all tissues, are the major macromolecular constituents of the ECM. GAGs significantly influence angiogenesis, proliferation, invasion, and metastasis of cancer cells. Accumulation of hyaluronan, a key GAG component of ECM, occurs in many solid tumors and is associated with poor prognosis and resistance to therapy across several malignancies. Hyaluronan may act as a signaling mediator by binding with hyaluronic acid receptors such as CD44, the hyaluronan-mediated motility receptor (RHAMM), Toll-like receptors 2 and 4, lymphatic vessel endothelial hyaluronan receptor (LYVE-1) and hyaluronan receptor for endocytosis (HARE)/stabilin-2. These interactions influence multiple intracellular signaling pathways, notably ERBB2 activation and anti-apoptotic mechanisms (Afratis et al., 2012). Enzymatic degradation of hyaluronan using PEGylated recombinant human hyaluronidase (PEGPH20) has been shown to enhance the uptake of checkpoint inhibitors in the TME, increase infiltration of CD8^+^ T cells and NK cells into tumor tissue, reduce MDSC numbers, and lead to improved tumor growth suppression *in vivo* (Singha et al., 2015; Clift et al., 2019).

Another crucial GAG component in the ECM is heparan sulfate (HS), whose chains covalently bind to various core proteins to form HS proteoglycans (HSPGs). HSPGs constitute the major structural components of the tumor ECM. NCRs and KIR2DL4 (CD158D) recognize HS chains in HSPGs on the cell surface, either on target cells (*-trans*) or on NK cells themselves (*-cis*). *Cis*-interaction of NCRs with the HSPGs (on the NK cells surface) can alter the surface distribution and function of the receptor by masking interactions with HS or other ligands on adjacent target cells (*trans*-interactions) (Hecht et al., 2009; Brusilovsky et al., 2014; Brusilovsky et al., 2015). Low heparanase expression in oral squamous cell carcinoma has been associated with increased infiltration of activated NK cells in the TME. Furthermore, inhibition of heparanase expression in breast cancer cells via knockdown of the bromodomain PHD finger transcription factor (BPTF), a known activator of heparanase expression, enhanced antitumor NK cell-mediated cytolytic activity in the TME (Mayes et al., 2017; Wang et al., 2023).

ECM components such as fibronectin, laminin, vitronectin and ECM proteoglycans are extracellular substrates of granzyme B released by cytotoxic lymphocytes. Granzyme B plays a key role in the induction of target cell death upon internalisation in the presence of perforin and also possesses potent ECM remodeling activity (Buzza et al., 2005; Boivin et al., 2012).

Due to their capacity to bind soluble extracellular proteins, ECM components, particularly fibronectin and GAGs, act as a reservoir of growth factors and cytokines. These bound factors are protected from degradation and exert localized effects on adjacent cells. TGF-β is among the most critical and abundant growth factor in the TME of solid tumors. TGF-β is a secreted pleiotropic cytokine that regulates cell proliferation, differentiation and apoptosis. Fibronectin immobilises latent TGF-β-binding protein-1 (LTBP-1), thereby retaining TGF-β in the ECM (Klingberg et al., 2018). Dysregulated secretion and activation of extracellular TGF-β stimulates fibroblasts to accumulate rigid and disordered ECM, resulting in fibrosis.

TGF-β also contributes significantly to immune suppression by promoting Treg cell proliferation and suppressing the proliferation and function of effector T cells, antigen-presenting DCs and NK cells. Additionally, TGF-β regulates the complex behavior of macrophages and neutrophils, thereby creating a negative immune regulatory network (Batlle and Massagué, 2019). For example, it has been established that the release of myeloid cells, and in particular neutrophils, from hematopoietic organs and reservoirs is influenced by tumor-derived factors, in particular, chemokines (Ugel et al., 2015). Aberrantly elevated expression of the corresponding CXC chemokines (from CXCL1 to CXCL3 and from CXCL5 to CXCL8) containing the conserved Glu-Arg-Leu motif (ELR), which are considered intermediate target signals for neutrophil chemotaxis, has been found in various solid tumors (Zou et al., 2014; SenGupta et al., 2021). Neutrophils recruited by the chemokine gradient in TME generate neutrophil extracellular traps (NETs) which inhibit CTL access to cancer cells and release specific matrix-remodeling enzymes such as neutrophil elastase and MMPs, thereby creating a permissive environment for tumor growth and dissemination (Awasthi and Sarode, 2024). As shown in an inducible mouse model of colon tumor, neutrophils secrete MMP9 (Germann et al., 2020), which subsequently proteolytically cleaves and activates the latent TGF-β deposited in the TME. TGF-β, in turn, skews the polarization of nonactivated neutrophils and macrophages towards phenotypes similar to N2 (tumor-associated neutrophils, TANs) and similar to M2 (tumor-associated macrophages, TAMs), respectively (Fridlender et al., 2009; Zhang et al., 2016).

In NK cells, TGF-β downregulates the expression of IFN-γ and the transcription factor T-Bet/TBX21, both of which are essential for NK cell development, maturation and function (Li and Sun, 2018; Huang and Bi, 2021; Lian et al., 2021). TGF-β induces the dedifferentiation of NK cells into innate lymphoid cell-1 (ILC1)-like phenotypes with impaired effector function (Gao et al., 2017; Berrien-Elliott et al., 2023). TGF-β also upregulates the expression of chemokine receptors, CXCR3 and CXCR4, characteristic of immature CD56brightCD16- NK cells, and inhibits CX3CR1 expression in mature CD56dimCD16+ NK cells, leading to reduced cytotoxicity (Hamann et al., 2011; Castriconi et al., 2013; Cózar et al., 2021). Additionally, TGF-β suppresses the expression of activating receptors such as NKG2D, NKp30 (Castriconi et al., 2003) and NKp44 and disrupts perforin polarisation at the immune synapse, thereby further impairing NK cell cytotoxicity (Donatelli et al., 2014). Donatelli et al. also demonstrated that TGF-β induces the production of microRNA-183, which inhibits the transcription and translation of DNAX activating protein 12 kDa (DAP12), a protein crucial for the stabilisation of surface NK receptors and subsequent signal transduction. Neutralisation of TGF-β1 with monoclonal antibodies can completely restore NKG2D expression and cytotoxic functions of NK cells (Lee et al., 2004). Suppression of TGF-β signal transduction in NK cells has been shown to enhance their anti-metastatic potential in murine tumor models without affecting NK cell development or homeostasis (Viel et al., 2016). At the same time, as shown by Thangaraj et al., iPSC-derived NK cells engineered to resist TGF-β signaling showed markedly improved persistence and efficacy in an aggressive mouse model of hepatocellular carcinoma (Thangaraj et al., 2024).

Of the many signaling pathways that modulate NK cell activity in the TME, the PI3K-AKT-mTORC1 signaling axis is of particular significance. The mTOR-mediated cellular metabolism is involved in the interaction of cancer and immune cells with TME during tumor progression. On the one hand, mTOR not only mediates mechanotransduction during changes in ECM stiffness, but also regulates the degradation of ECM proteins as an alternative metabolic pathway. When tumor epithelial cells are deprived of amino acids, repression of mTOR rearranges cellular metabolism to utilize amino acids derived from ECM protein degradation, to the benefit of nutrient-starved cells (Colombero et al., 2021). On the other hand, mTOR activity is required for the glycolytic reprogramming of activated NK cells, and this metabolic shift is a prerequisite for normal NK cell effector functions, such as IFN-γ production and increased granzyme B expression (Donnelly et al., 2014). TGF-β and the mTOR inhibitor rapamycin reduce NK cell metabolic activity and proliferation as well as the expression of various NK cell receptors, thereby diminishing their cytotoxic capacity (Marçais et al., 2014; Viel et al., 2016). Constitutive TGF-β signaling or mTOR depletion delays NK cell development, whereas deletion of the TGF-β receptor subunit TGF-βRII enhances mTOR activity and NK cell cytotoxicity in response to IL-15, which activates PI3K-AKT-mTORC1 signaling and promotes T-Bet expression in NK cells (Huang and Bi, 2021). At the same time, it should be noted that direct manipulation of the tumor ECM, for example, through blockade of TGF-β or MMPs, may prove unsuccessful due to serious side effects associated, among others, with disruption of normal tissue homeostasis (Krüger et al., 2010; Colak and ten Dijke, 2017; Winer et al., 2018; Teixeira et al., 2020).

## 5 Conclusion

Today, immunotherapy is one of the most advanced areas in basic research and clinical practice. An analysis of ongoing clinical trials investigating NK cell-based immunotherapy for solid tumors reveals its substantial potential (Jørgensen et al., 2025; Wu et al., 2025). Researchers note that most of the clinical trials of NK cell-based drugs launched worldwide are based on unmodified NK cells (71.4%), while only a minority involve CAR-NK cells (28.6%). Although researchers emphasize that TME has a serious immunosuppressive effect on NK cells, current CAR-NK design strategies are primarily focused on enhancing cytotoxicity alone (Wu et al., 2025).

The pivotal role of ECM stiffness and mechanotransduction signaling pathways in the success and prognosis of cancer immunotherapy, as well as in the accuracy of immunotherapeutic drug delivery, is now widely recognised. Approaches to overcome the TME remain experimental. In particular, it is proposed to use photothermal therapy to reduce the density of TME (Li J. et al., 2024). Efforts to modify ECM stiffness, including the development of biomaterials that more closely mimic native ECM, will facilitate a deeper understanding of how ECM mechanics regulate immune cell activity in solid tumors, ultimately leading to the development of novel therapeutic strategies (Zhang et al., 2024; Du et al., 2024; Miao et al., 2024; Lightsey and Sharma, 2024; Mai et al., 2024).

Current strategies aim both to overcome tumor evasion of the immune response by enhancing NK cell recognition and cytotoxicity, and to promote immune cell persistence, infiltration and resistance to the TME (Page et al., 2024). Combining NK cell transfer with conventional therapies such as chemotherapy and immunotherapy can significantly improve outcomes (Fanijavadi et al., 2025). A synergistic effect has been observed when CAR-NK cells are used alongside monoclonal antibodies targeting either activating receptors such as CD16, immune checkpoint inhibitors such as PD-L1 and PD-1, or tumor antigens. In addition, it seems reasonable to use monoclonal antibodies and the recombinant proteins mentioned above targeting TME components in combination with NK cell therapy. Novel genetic modifications of NK cells to express chemokine receptors that enhance their chemotropism, or to produce cytokines or cytokine receptors that facilitate TME penetration, are also promising strategies for improving therapeutic efficacy (Ran et al., 2022).

These emerging strategies underscore the growing sophistication of adaptive cell therapy aimed at reprogramming NK cells to overcome the challenges posed by the immunosuppressive TME and to stimulate robust anti-tumor immune response. Enhancing the tumor-targeting capabilities of adoptively transferred NK cells countering their suppression and depletion within the TME, improving their persistence in allogeneic settings, and increasing their long-term immune surveillance in cases of tumor recurrence are the current goals of NK cell therapy.

In this context, it is appropriate to reiterate the simple “3C rule” previously formulated and potentially beneficial for NK cell-based immunotherapeutic approaches to solid tumors (Kuznetsova et al., 2024). In brief,

1. Consolidate various analytical procedures, including histological subtyping, and molecular genetic profiling, etc., to predict individual patient responses to treatment.
2. Crack the defence systems of both cancer cells and the TME by simultaneously targeting the malignant cells themselves, cancer-associated fibroblasts and ECM components.
3. Cogitate on/combine the strategic use of multiple CAR-immune cell types (e.g., CAR-T, CAR-NK, CAR-M, CAR-NKT), administered in parallel or sequentially. This combinatorial approach is expected to compensate for the limitations of individual cell types and elicit an immune response that closely approximates natural immunity.

Recent advances in understanding the basic biology of NK cells and genetic manipulation techniques, suggest a splendid future for NK cell immunotherapy not only in cancer treatment but also in addressing autoimmune diseases and infections. Interdisciplinary collaboration integrating oncology, cell biology, physics, engineering, materials science and nanotechnology are crucial in advancing therapeutic strategies targeting ECM rigidity and mechanotransduction signaling pathways.
